# Supplementary material for: 3D Ultrasensitive Polymers-Plasmonic Hybrid Flexible Platform for In-Situ Detection
Source: Polymers (Basel). 2020 Feb 9;12(2):392. doi: 10.3390/polym12020392 (PMC7077657; doi:10.3390/polym12020392)
Supplement: Supplementary file 1 [file polymers-12-00392-s001.pdf]

Supplementary Materials:

3D Ultrasensitive Polymers-Plasmonic Hybrid Flexible Platform for In-Situ Detection

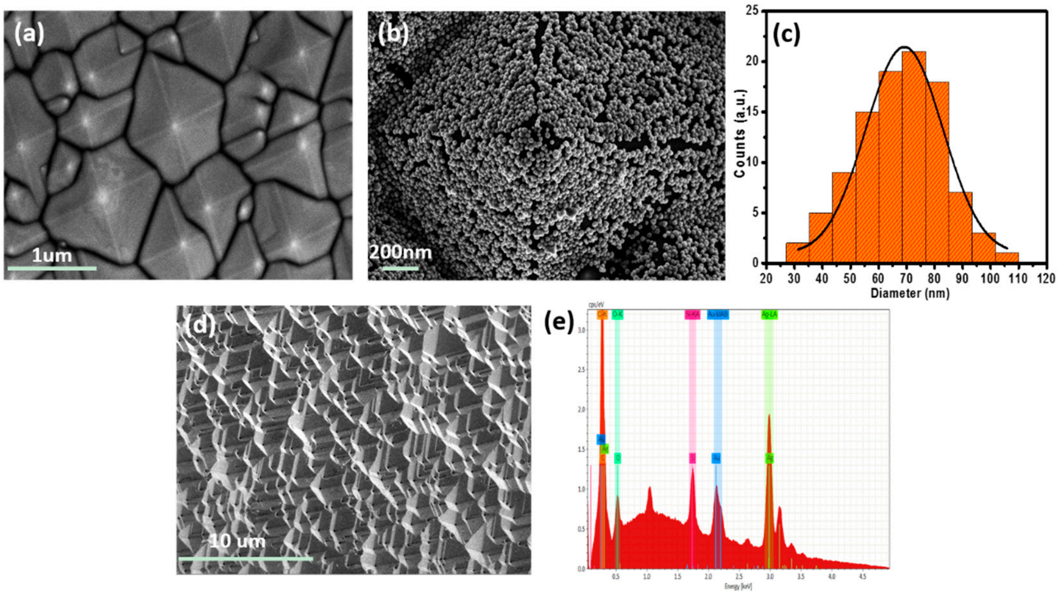

Figure S1. **(a)** SEM image of the P-Si sample; **(b)** SEM image of Ag NPs deposited on the P-Si substrates; **(c)** The size distribution of Ag NPs; **(d)** SEM images of the P-AgNPs@PMMA substrate in high magnification; **(e)** The corresponding EDS spectrum of the P-AgNPs@PMMA substrate.

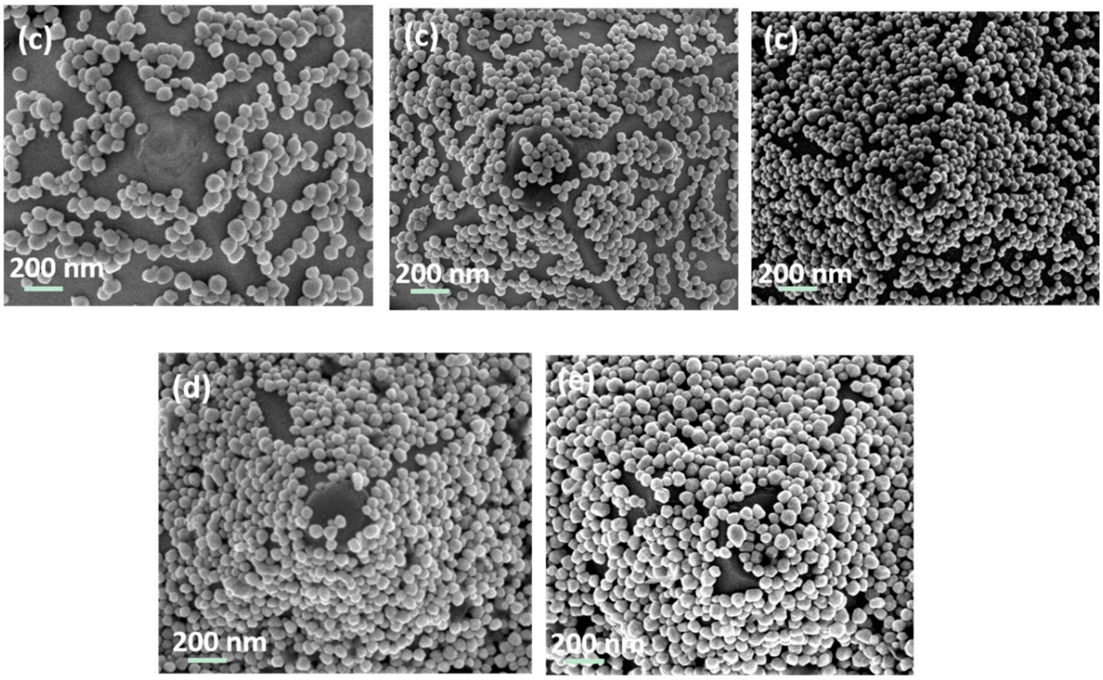

Figure S2. SEM images of different volume ratio AgNPs@PMMA/P-Si substrate. **(a)** PMMA/Ag = 2:1; **(b)** PMMA/Ag = 1:1; **(c)** PMMA/Ag = 2:3; **(d)** PMMA/Ag = 1:2 and **(e)** PMMA/Ag = 2:5.
